# Genesee Valley Veterinary Medical Association

**Published:** 1897-12

**Authors:** 


Secretary.

### THE GENESEE VALLEY VETERINARY MEDICAL ASSOCIATION.

### VETERINARIANS OF ROCHESTER AND MONROE COUNTIES OF NEW YORK STATE FORM A SOCIETY.

A meeting of the veterinary surgeons of Rochester and Monroe county was held at the Livingston Hotel on November 16th for the purpose of forming a permanent organization for mutual benefit and protection to the members. The following temporary officers were chosen: Dr. A. Drinkwater, Chairman; Dr. A. G. Tegg, Secretary, and Dr. L. R. Webber, Treasurer. After an extended discussion, it was decided to hold another meeting at the same place on Thursday of next week, to which an invitation to attend will be extended to the members of the profession in Monroe, Wayne, Orleans, Genesee, Livingston, and Ontario counties.

Those present at the meeting, other than the temporary officers mentioned above, were: Drs. Albert Tegg, John J. Tegg, J. C. McKenzie, I. W. Drinkwater, and E. Knight, of Rochester; Dr. O. B. French, of Honeoye Falls; Dr. Taylor, of Henrietta, and Dr. Palmer, of Scottsville.

The veterinary surgeons of Western New York held a second meeting at the Livingston Hotel on November 18th, 1897, and perfected an organization to be known as the Genesee Valley Veterinary Medical Association. Veterinarians were present from the surrounding towns as follows: Dr. A. Drinkwater, Rochester; Dr. W. G. Dodds, Canandaigua; Dr. A. George Tegg, Rochester; Dr. L. R. Webber, Rochester; Dr. Rich, Avon; Dr. French, Honeoye Falls; Dr. Taylor, Henrietta; Dr. Steiner, Bergen; Dr. Hill, Wolcott; Dr. Leffer, Genesee; Dr. Earl, Palmyra; Dr. Knight and Dr. R. Palmer, Scottsville; Dr. McKenzie, Rochester.

The following officers were elected: President, Dr. Drinkwater; Vice-President, Dr. Dodds; Secretary, Dr. Tegg; Treasurer, Dr. Webber; Censors, Drs. Rich, French, and Taylor.

The object of the association is to discuss the various questions of interest to veterinarians, and papers will be prepared by the different members and read before the association. The next meeting will be held at the Livingston Hotel, on Thursday, December 9th, when papers will be read by Drs. Steiner and Palmer.

